# Diversity of midgut bacteria in larvae and females of *Aedes aegypti* and *Aedes albopictus* from Gampaha District, Sri Lanka

**DOI:** 10.1186/s13071-021-04900-5

**Published:** 2021-08-28

**Authors:** Koshila Ranasinghe, Nayana Gunathilaka, Deepika Amarasinghe, Wasana Rodrigo, Lahiru Udayanga

**Affiliations:** 1 Department of Zoology and Environmental Management, Faculty of Science, University of Kelaniya, Dalugama, Sri Lanka; 2 Department of Parasitology, Faculty of Medicine, University of Kelaniya, Ragama, Sri Lanka; 3 Department of Zoology, Faculty of Natural Sciences, The Open University of Sri Lanka, Nawala, Nugegoda, Sri Lanka; 4 Department of Bio-Systems Engineering, Faculty of Agriculture and Plantation Management, Wayamba University of Sri Lanka, Makadura, Sri Lanka

**Keywords:** Midgut, Bacteria, Diversity, *16S rRNA* gene, Mosquitoes, *Aedes*

## Abstract

**Background:**

The midgut microbiota of mosquitoes maintain basal immune activity and immune priming. In recent years, scientists have focused on the use of microbial communities for vector control interventions. In the present study, the midgut bacteria of larvae and adults of *Aedes aegypti* and *Ae. albopictus* were assessed using both field-collected and laboratory-reared mosquitoes from Sri Lanka.

**Methods:**

Adults and larvae of *Ae. aegypti* and *Ae. albopictus* were collected from three selected areas in Gampaha Medical Officer of Health area, Gampaha District, Western Province, Sri Lanka. Bacterial colonies isolated from mosquito midgut dissections were identified by PCR amplification and sequencing of partial *16S rRNA* gene fragments.

**Results:**

Adults and larvae of *Ae. aegypti* and *Ae. albopictus* harbored 25 bacterial species. *Bacillus endophyticus* and *Pantoea dispersa* were found more frequently in field-collected *Ae. aegypti* and *Ae. albopictus* adults, respectively. The midgut bacteria of *Ae. aegypti* and *Ae. albopictus* adults (*X*^2^ = 556.167, *df* = 72, *P* < 0.001) and larvae (*X*^2^ = 633.11, *df* = 66, *P* < 0.001) were significantly different. There was a significant difference among the bacterial communities between field-collected adults (*X*^2^ = 48.974, *df* = 10, *P* < 0.001) and larvae (*X*^2^ = 84.981, *df* = 10, *P* < 0.001). *Lysinibacillus sphaericus* was a common species in adults and larvae of laboratory-reared *Ae. aegypti.* Only* P. dispersa *occurred in the field-collected adults of* Ae. aegypti* and* Ae. albopictus*. Species belonging to genera *Terribacillus*, *Lysinibacillus*, *Agromyces* and *Kocuria* were recorded from *Aedes* mosquitoes, in accordance with previously reported results.

**Conclusions:**

This study generated a comprehensive database on the culturable bacterial community found in the midgut of field-collected (*Ae. aegypti* and *Ae. albopictus*) and laboratory-reared (*Ae. aegypti*) mosquito larvae and adults from Sri Lanka. Data confirm that the midgut bacterial diversity in the studied mosquitoes varies according to species, developmental stage and strain (field* vs* laboratory).

**Graphical abstract:**

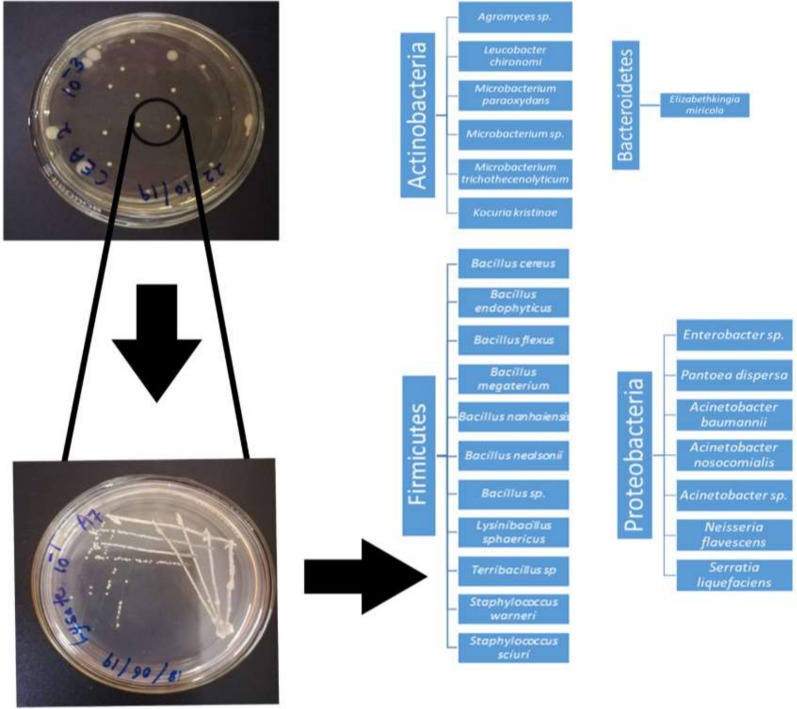

**Supplementary Information:**

The online version contains supplementary material available at 10.1186/s13071-021-04900-5.

## Background

Mosquitoes have received more attention than other arthropod vectors because of their ability to transmit many pathogens causing significant diseases, including malaria, dengue, zika and chikungunya. The increasing incidence of dengue virus (DENV) and other arboviruses worldwide is a major public health concern [1]. *Aedes aegypti* and *Aedes albopictus* are highly anthropophilic species that are responsible for the transmission of DENV and other arboviruses [2, 3].

Conventional mosquito control measures have many downsides, such as the development of insecticide resistance, which have led to efforts to develop novel strategies needed for an integrated vector management (IVM) approach [4]. These efforts have resulted in the development of the sterile insect technique (SIT) [5] and incompatible insect technique (IIT) [5, 6] as novel strategies. However, additional methods for mosquito control are still urgently needed [2].

In recent years, scientists have investigated the potential use of microbial communities for vector control interventions. The latest studies have emphasized the importance of the microbial community to maintain basal immune activity and immune priming in insects [7, 8]. In addition to stimulating the mosquito immune responses, bacteria can also influence mosquito competence by impairing pathogen infection through competition for resources or secretion of anti-pathogenic molecules [9, 10]. As a mosquito control strategy, this method strategy involves genetically modifying symbiotic bacteria to express effector molecules and then reintroducing these modified bacteria into mosquitoes, where they produce the desired effect by manipulating the transmission potential of the targeted disease [11]. The procedure is referred to as paratransgenesis and has proven to be a highly valuable tool for mosquito control [11].

Mosquitoes acquire microbial communities through vertical inheritance and from the surrounding environment [12]. The midgut microbiota in mosquitoes is assimilated by aquatic larval stages and by nectar- or blood-feeding during the adult stages. For example, native breeding sites where mosquito larvae feed and grow may influence the composition of the gut microbiota [13, 14]. Some studies have highlighted that environmental disturbance can reduce diversity in the gut microbiota, leading to the hypothesis that gut microbiota diversity can be used to measure the fitness of a species invading a new habitat [15].

In some modern mosquito control strategies, genetically modified mosquitoes are artificially reared and subsequently released into the environment, with the aim to reduce vector densities through population replacement or suppression. One factor that needs to be considered, however, is that the colonization of new habitats by a genetically modified species could be a challenge to that species due to reduced fitness [15]. In blood-feeding insect vectors, microbiota plays another vital function by affecting the competence of the vector to transmit pathogens to their susceptible host. Therefore, monitoring the gut microbial community in a modified mosquito strain can represent a different approach in terms of vector competence and fitness since the occurrence of different microbiota directly affects the immunity, fitness and survival of the insects [16].

Therefore, it could be useful to incorporate genetic modification with the gut microflora of modified strains in a strategy to generate an organism compatible with the wild population, thereby achieving higher competitiveness, fitness and survival of the modified mosquito. Further, studying the gut microbiota would be beneficial for identifying potential microbial candidates and exploring the feasibility of using some of these bacteria in novel vector control strategies such as paratransgenesis.

In Sri Lanka, new mosquito control approaches for dengue control, such as the use of SIT, IIT and modified vectors, have been initiated [2]. However, no attempt has yet been made to study the bacterial community in the midgut of *Aedes* mosquitoes. Hence, the present study was aimed at determining the composition of the midgut bacteria of larvae and adults of *Ae. aegypti* and *Ae. albopictus*, using both field-collected and laboratory-reared mosquitoes from Sri Lanka.

## Methods

### Selection of study area

Sri Lanka is a tropical island located in the Indian Ocean, next to the southern tip of India. On the basis of topography, the island can be mainly divided into central highlands and surrounding lowlands, with the central highlands including complex topographical features, such as ridges, peaks, basins, valleys and plateaus. The surrounding lowlands are generally flat except for small hills located in several places [17].

Mosquito surveys were conducted from May 2018 to August 2019 at three selected sites, namely in the vicinities of Brandiyamulla (07°079ʹN, 80°016ʹE), Gampaha (07°092ʹN, 79°993ʹE) and Miriswaththa (07°073ʹN, 80°012ʹE) in the Gampaha Medical Office of Health (MOH) area, Gampaha District (7°08ʹN, 80°00ʹE), Western Province of Sri Lanka. The average elevation of Gampaha District is 32.9 m a.s.l. This district lies in the south-western lowland wet climatic zone and has an average annual temperature of 27.3 °C (range 25–32.5 °C) and annual mean rainfall that ranges from 2000 to 3500 mm [18]. Gampaha District harbors an array of natural and human-influenced forest and wetland ecosystem types. In recent years, this district is also the second high-risk district in Sri Lanka for dengue [19].

The MOH area in the district has been identified and systematically surveyed for dengue vector mosquitoes with the aim to implement SIT-based control approaches using irradiated males and a locally developed strain of *Wolbachia* triple-infected mosquitoes [2, 20, 21]. To accomplish this, surveillance sites for mosquito collections were purposely selected from the Gampaha District to collect the fundamental data required for a number of novel vector control interventions.

### Mosquito collection

Adult mosquitoes were collected using a Prokopack aspirator (John W. Hock Company, Gainesville, FL, USA) at outdoor resting sites, and larvae were collected using the siphoning/pipetting method from container breeding habitats following the guidelines described by the World Health Organization [22]. Both the sexes of the two species targeted were collected and used in subsequent experiments. Field-collected adult mosquitoes were transferred intact to adult rearing cages while the collected larvae were transferred safely into larval rearing containers. All collected mosquito larvae and adults were transported to the insectary facility of the Department of Parasitology, Faculty of Medicine, University of Kelaniya, Ragama, Sri Lanka. Field samples were processed (see section Processing of mosquitoes samples) within 1–2 h after collection.

### Laboratory-reared colony of *Ae. aegypti*

For comparison with the midgut microbiota of field-collected mosquitoes, we used larvae and adults from a laboratory colony of *Ae. aegypti* (F10 generation) reared under standard conditions (26 ± 1 °C and 75–80% relative humidity) at the Department of Parasitology, Faculty of Medicine, University of Kelaniya. The larvae were fed twice daily (08:30 h and 15:30 h) with a larval diet containing tuna meal, bovine liver powder and brewer’s yeast, which was optimized for larval rearing at the insectary [23]. Adults were housed in mosquito rearing cages (24 × 24 × 24 cm^3^) with mesh screening and provided with a 10% sugar solution and water ad libitum. Every 3 days, the female mosquitoes were fed for 30 min with bovine blood using the artificial metal plate technique [23].

### Processing of mosquitoes samples

Adult mosquitoes were killed by cold shock, followed by separation based on key morphological characteristics [24, 25]. Stage III and IV larvae were sacrificed for the experiment. The specimens were surface-sterilized individually for 30 s in a microcentrifuge tube containing 250 µl of 70% ethanol followed by two rinses in 250 µl of phosphate-buffer saline (PBS). The final discard was cultured for bacteria screening to confirm that there was no contamination. No bacterial growth was noted in the discards.

The midgut of female mosquitoes and larvae (stages III and IV) were dissected under sterile conditions under a dissecting binocular microscope (Lebomed CZM4 Binocular Zoom Stereo Microscope; Labo America Inc., Freemont, CA, USA). The dissected midgut of each mosquito was individually transferred to a 1.5-ml microcentrifuge tube containing 250 μl of PBS and homogenized with a sterilized micropestle. The homogenized lysate was serially diluted in PBS (900 μl) to prepare a serial dilution from 10^0^ to 10^–7^. A minimum sample size of 250 females and larvae was screened for midgut bacteria in each mosquito species, either field-collected or laboratory-reared.

### Bacteria isolation from plate cultures

A 100-µl volume from each dilution was plated on sterile plate count agar (PCA) and incubated at 35 °C for 24–48 h. Microbial growth was assessed based on the total number of colony-forming units (CFUs). Bacterial colonies were distinguished morphologically (i.e. shape, size, color, margin, opacity and elevation). Morphologically distinct colonies were selected from primary plates for repeated subculture on nutrient agar plates until a pure colony was obtained.

### Confirmation of bacterial isolates from genomic sequencing

Bacterial DNA was extracted from each pure culture using the QIAamp DNA Mini Kit (QIAGEN GmbH, Hilden, Germany), following the manufacturer’s guidelines. PCR amplifications of the partial *16S rRNA* gene sequence were performed using universal primers 27F (5′-AGAGTTTGATCCTGGCTCAG-3′) and 1492R (5′-TACGGCTACCTTGTTACGACTT-3′) [26]. PCR analyses were performed with a reaction mixture containing 1× PCR buffer (Invitrogen™, Thermo Fisher Scientific, Waltham, MA, USA) , 0.5 μM of each primer, 2.5 mM MgCl_2_, 200 ng of purified DNA, 0.2 mM dNTPs and 0.3 units of *Taq* polymerase (Invitrogen™). The total volume was adjusted to 25 μl. PCA media and doubled-distilled water were used as negative controls.

The PCR cycling program consisted of an initial denaturation at 94 °C, 10 min; then denaturation at 94 °C/30 s, annealing at 55 °C/30 s and extension at 72 °C/1 min for 35 cycles; and a final extension at 72 °C for 8 min. The amplified product was visualized in a 1% agarose gel containing ethidium bromide using a UV transilluminator. The PCR amplicons were then purified using the QIAquick PCR Purification Kit (QIAGEN GmbH). The purified products were sent to Macrogen (Macrogen Inc., Seoul, Republic of Korea) for sequencing of the *16S rRNA* partial gene by the Sanger method.

### Phylogenetic analysis of midgut bacteria isolated from mosquito species

Homologous sequences were searched in the GenBank database using the Basic Local Alignment Search Tool (BLAST) [27]. The isolates were identified when their *16S rRNA* gene sequences shared 97% homology with the reference sequences. The evolutionary history was inferred using the neighbor-joining method. The evolutionary analyses were conducted in MEGA X. The evolutionary distances, computed using Tajima–Nei method, were used to infer the phylogenetic tree.

### Diversity of the bacterial community

An online calculator [28] was used to calculate 95% confidence intervals (CIs) for comparison of the presence of each bacterial species in each mosquito species. The significance in the distribution of bacteria species in *Ae. aegypti* and *Ae. albopictus* was evaluated using the Chi-square test, with a *P* < 0.05 being considered statistically significant.

To highlight the differences in the midgut microbiota in field-collected and laboratory-reared mosquitoes, the distance-based redundancy analysis (dbRDA) by Bray–Curtis (BC) dissimilarity [29] was used.

## Results

### Bacterial diversity in field-collected adult mosquitoes

**Fig. 1:**
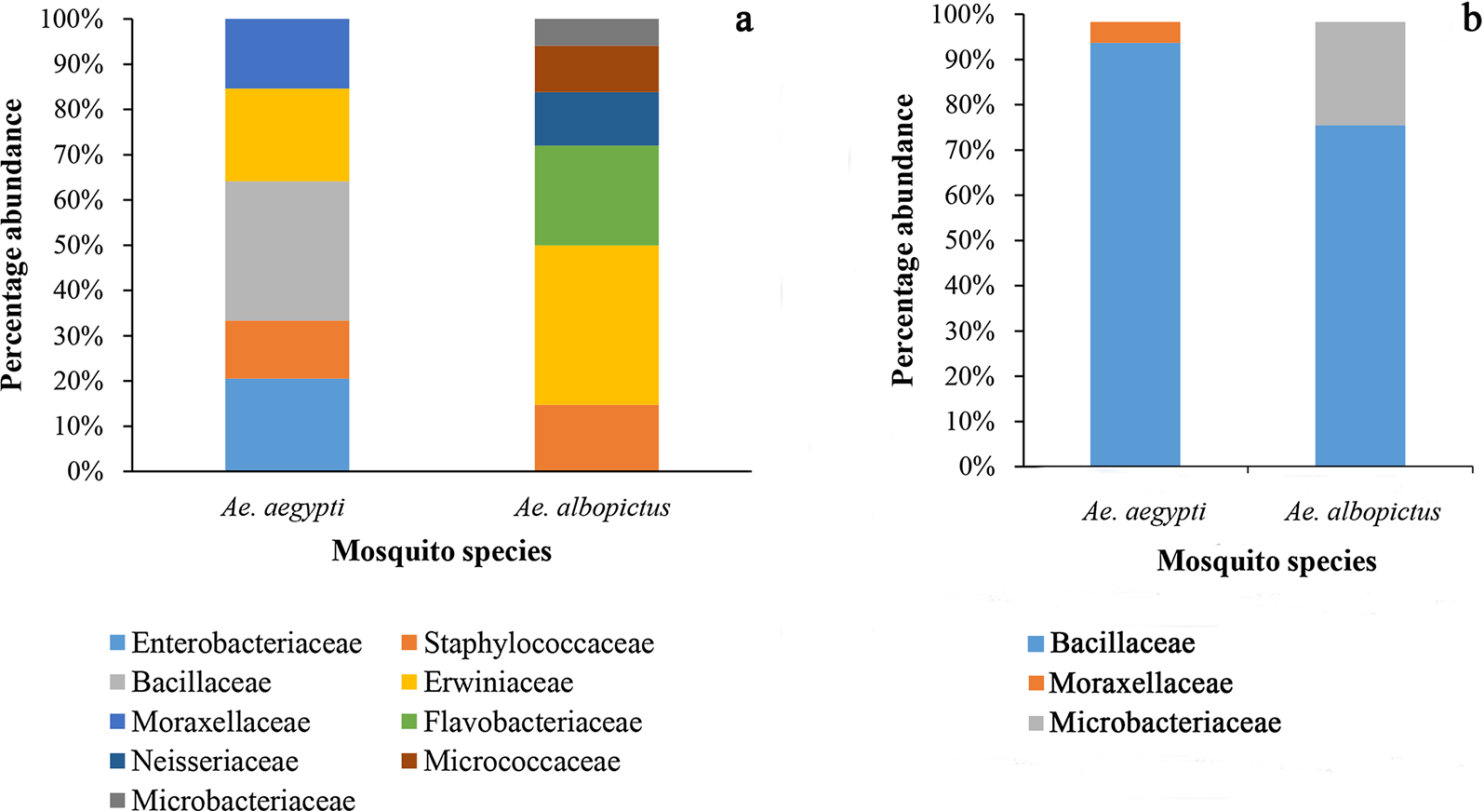
Relative abundance of bacterial families molecularly identified (*16S rRNA* gene sequence analysis) in the midgut of *Aedes aegypti* and *Ae. albopictus* collected in the field in Gampaha District, Sri Lanka. **a** Adults, **b** larvae

In terms of the number of bacterial colonies growing in the PCA culture medium, we observed a decreasing trend with increasing dilution of the culture medium in both adults and larvae of each mosquito species (Additional file 1: Figure S1). A better separation of colonies was observed at the 10^–3^ dilution (Additional file 1: Figure S1). Six bacterial strains were identified from field-collected *Ae. aegypti* and *Ae. albopictus* adults (Additional file 2: Table S1). The midgut bacteria identified in the field-collected *Ae. albopictus* adults belonged to six families (Staphylococcaceae, Erwiniaceae, Flavobacteriaceae, Neisseriaceae, Micrococcaceae and Microbacteriaceae). In comparison, midgut bacteria belonging to five families (Enterobacteriaceae, Staphylococcaceae, Bacillaceae, Erwiniaceae, and Moraxellaceae) were observed in field-collected *Ae. aegypti* adults (Fig. 1a). *Bacillus endophyticus* and *Pantoea dispersa* were the most common bacterial species isolated from *Ae. aegypti* and *Ae. albopictus* adults, respectively (Additional file 2: Table S1).

### Bacterial diversity in field-collected mosquito larvae

Bacteria belonging to the family Bacillaceae predominated in field-collected larvae of both *Ae. aegypti* (95.4%) and *Ae. albopictus* (76.8%). Species belonging to bacterial families Moraxellaceae and Microbacteriaceae were detected in larvae of *Ae. aegypti* and *Ae. albopictus*, respectively (Fig. 1b). Among the bacterial species recorded in mosquito larvae, *Bacillus flexus* predominated in *Ae. aegypti* and *Bacillus megaterium* perdominated in *Ae. albopictus* (Additional file 2: Table S1).

### Bacterial diversity in laboratory-reared *Ae. aegypti*

**Fig. 2:**
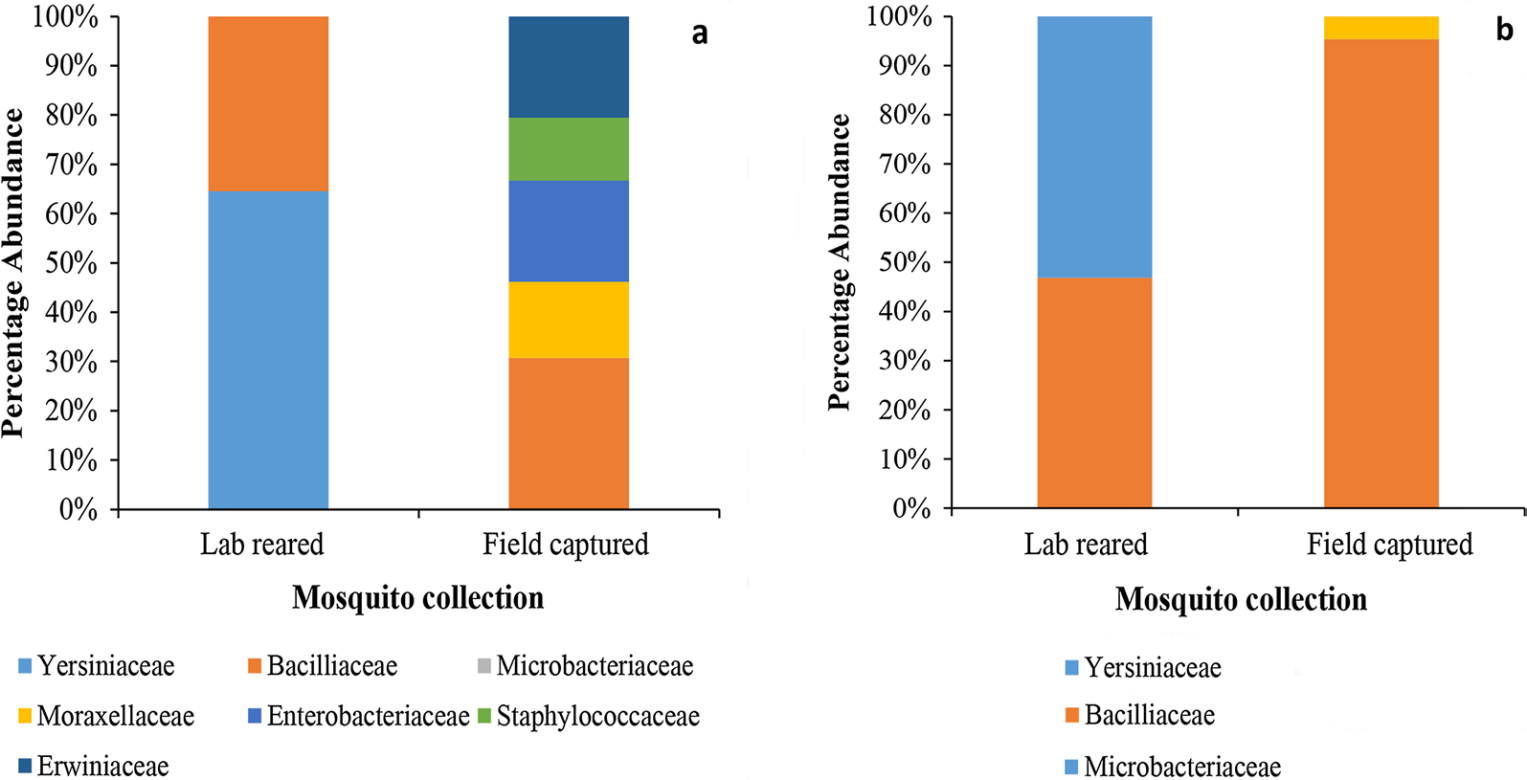
Relative abundance of bacterial families molecularly identified (*16S rRNA* gene sequence analysis) in the midgut of field-captured mosquitoes and laboratory-reared mosquitoes (Gampaha District, Sri Lanka). **a** Adults, **b** larvae

Laboratory-reared *Ae*. *aegypti* larvae harbored only two bacterial families (Microbacteriaceae and Bacillaceae) (Fig. 2). Similarly, only two bacterial species, namely *Serratia liquefaciens* (64.5%) and *Lysinibacillus sphaericus* (35.5%), were identified in laboratory-reared *Ae*. *aegypti* adults (Additional file 2: Table S1).

### Bacterial communities of field-collected versus laboratory-reared mosquitoes

Overall, the midgut microbiota of adult *Ae. aegypti* and *Ae. albopictus* mosquitoes (*X*^2^ = 556.167, *df* = 72, *P* < 0.001) and *Ae. aegypti* and *Ae. albopictus* larvae (*X*^2^ = 633.11, *df* = 66, *P* < 0.001) were significantly different. Also, the relative distribution of midgut microbiota differed significantly among field-collected adults (*X*^2^ = 48.974, *df* = 10, *P* < 0.001) and larvae (*X*^2^ = 84.981, *df* = 10, *P* < 0.001) collected from the three different study sites. A significant difference was also observed between laboratory-reared and field-collected adults (*X*^2^ = 194.265, *df* = 21, *P* < 0.001).

**Fig. 3:**
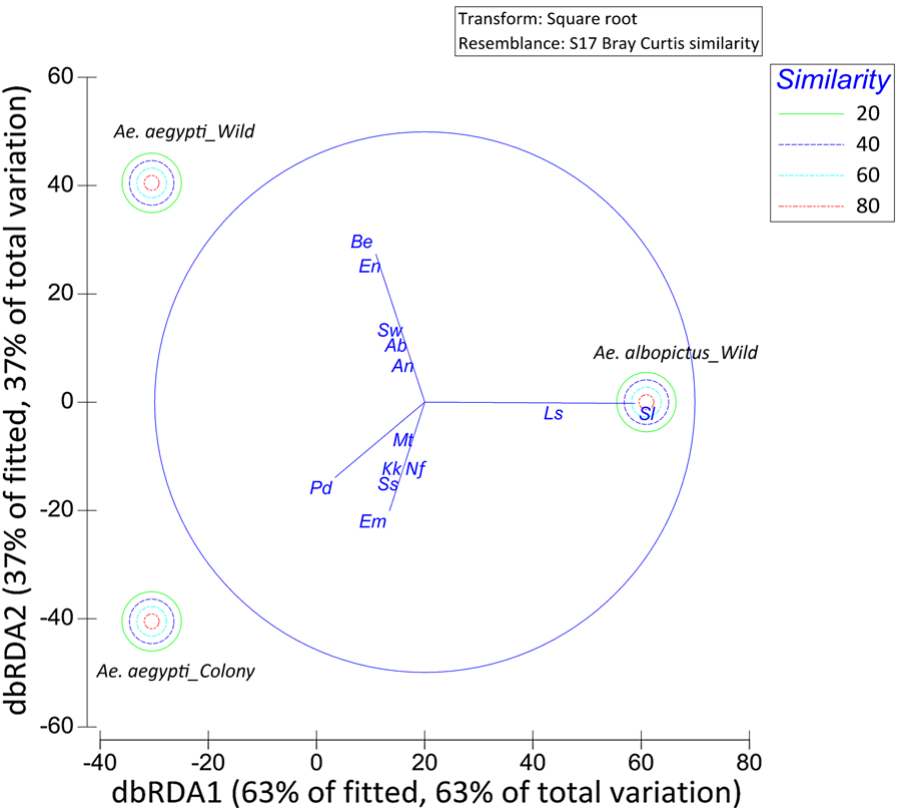
The distance-based redundancy analysis (*dbRDA*) plot for distribution of midgut bacteria in field-collected *Ae. aegypti* and *Ae. albopictus* adults and in laboratory-reared *Ae. aegypti* adults. *Mt* *Microbacterium trichothecenolyticum*,* Kk* *Kocuria kristinae*,* Em* *Elizabethkingia miricola*,* Be* *Bacillus endophyticus*,* Ls* *Lysinibacillus sphaericus*,* Sw* *Staphylococcus warneri*,* Ss* *Staphylococcus sciuri*,* En* *Enterobacter *sp., *Pd* *Pantoea dispersa*,* Ab* *Acinetobacter baumannii*,* An* *Acinetobacter nosocomialis*,* Nf* *Neisseria flavescens*,* Sl* *Serratia liquefaciens*

Among the total variations observed in the midgut bacteria, the dbRDA 1 and dbRDA 2 axes accounted for nearly 63 and 37% of variations, suggesting a good fit (Fig. 3). As indicated by the loadings of the dbRDA axes, the midgut microbiota of *Ae. aegypti* and *Ae. albopictus* adults had a similarity of 18.4%. No similarity was detected between the field-collected and laboratory-reared *Ae. aegypti* adults. The dbRDA 1 axis was significantly influenced by the abundance of *Serratia liquefaciens* and *Lysinibacillus sphaericus*, whereas the dbRDA 2 axis was significantly influenced by the abundance of *Bacillus endophyticus*, *Staphylococcus warneri* and *Enterobacter* sp.

**Fig. 4:**
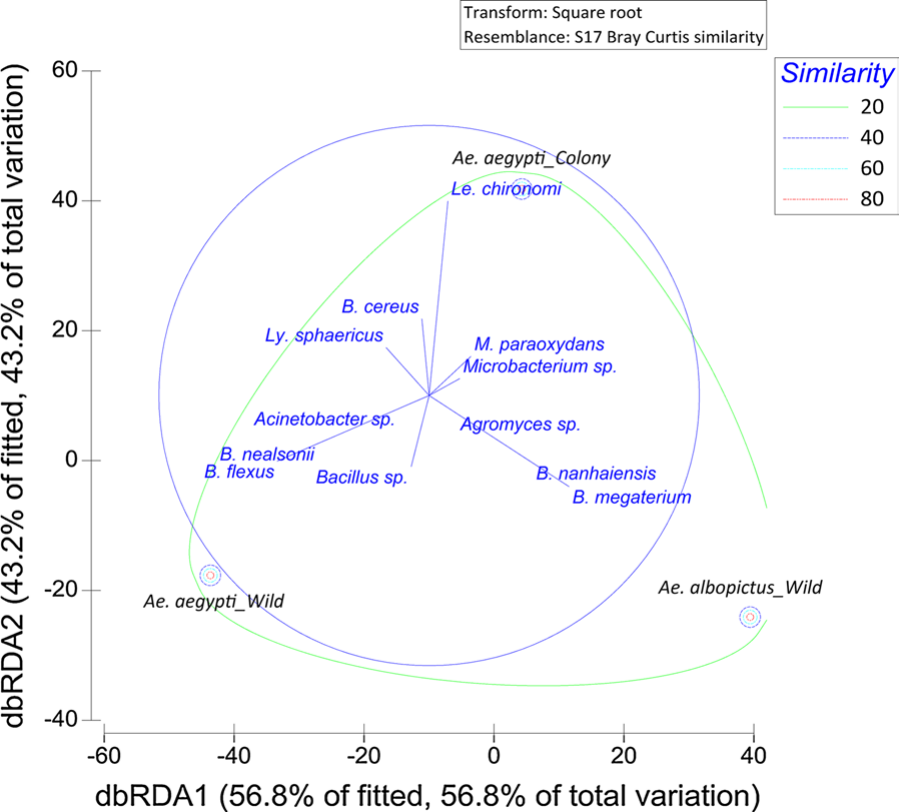
The dbRDA plot for distribution of midgut microbiota in larvae from field-collected *Ae. aegypti* and *Ae. albopictus* and laboratory-reared *Ae. aegypti* larvae

The relative distribution of midgut microbiota in field-collected and laboratory-reared larvae of *Ae. aegypti* differed significantly (*X*^2^ = 222.519, *df* = 24, *P* < 0.01). Midgut microbiota harbored by laboratory-reared *Ae. aegypti* larvae formed a separate sub-cluster based on the BC similarity (Fig. 4). However, as indicated by the loadings of the dbRDA axes, the midgut microbiota of laboratory-reared *Ae. aegypti* larvae shared a similarity of 24.6 and 22.7% with the those of field-collected *Ae. aegypti* and *Ae. albopictus* larvae, respectively. In addition, the midgut microbiota of field-collected larvae of *Ae. aegypti* and *Ae. albopictus* also had a similarity of 19.0%. The dbRDA 1 axis was significantly influenced by the abundance of *Bacillus flexus*, *B. nealsonii* and *Lysinibacillus sphaericus*, while the dbRDA 2 axis was significantly influenced by the abundance of *Leucobacter chironomy* and *Bacillus cereus.*

The diversity of bacteria recorded from the phylum Bacteroidetes was comparatively lower than that recorded from the other three phyla (Proteobacteria, Actinobacteria and Firmicutes). As indicated by the loadings of dbRDA axes (Fig. 4), the presence of *Bacillus* spp. in both *Ae. aegypti* and *Ae. albopictus* larvae accounted for the similarity of 23.5% between these mosquito species. Meanwhile, the presence of *Pantoea dispersa* in *Ae. aegypti* and *Ae. albopictus* adults accounted for the similarity of 19% between these mosquito species.

### Phylogenetic analysis inferred by *16S rRNA* gene sequences from bacterial isolates

**Fig. 5:**
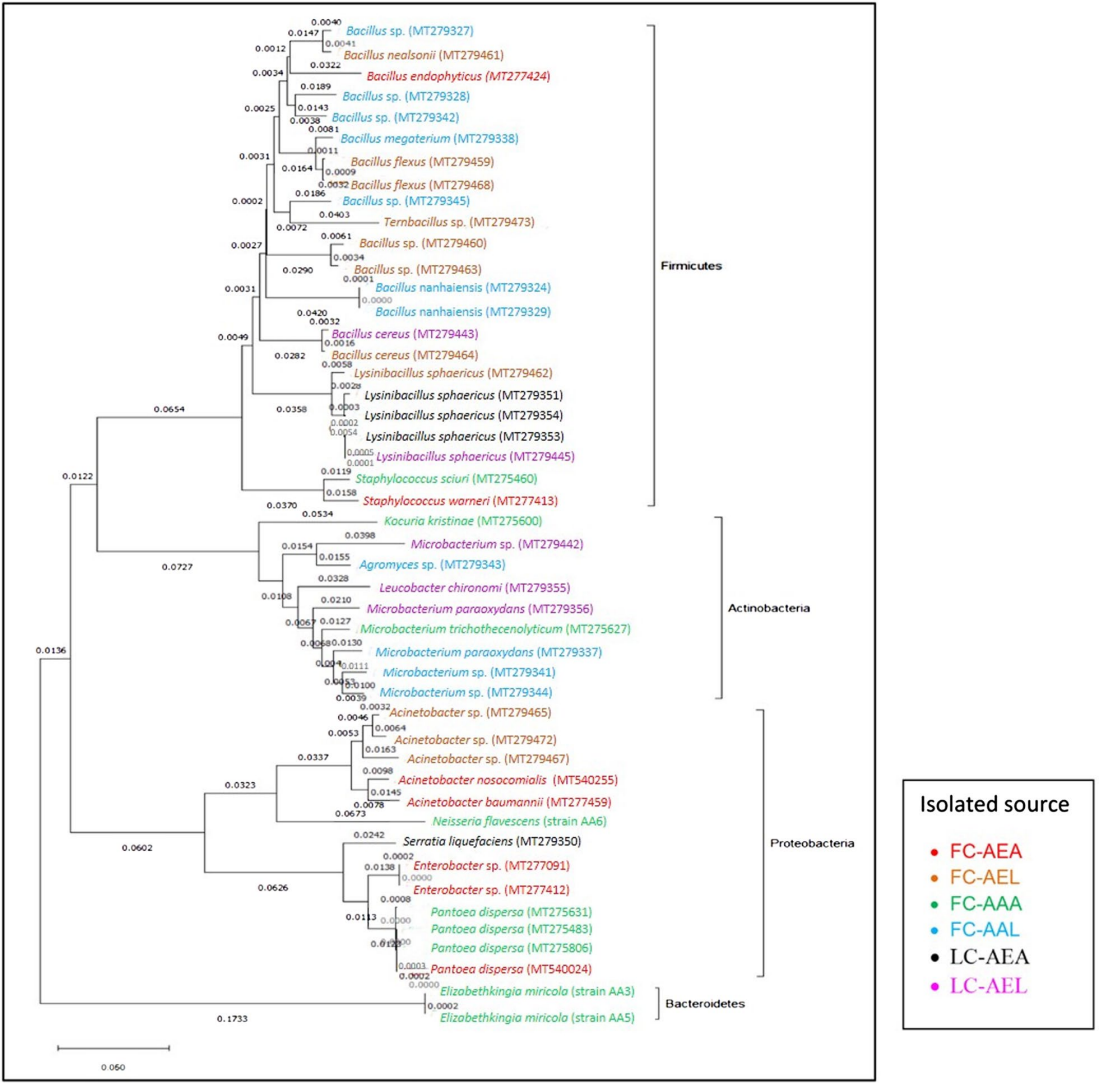
Phylogenetic tree of *16S rRNA* gene sequences from bacterial isolates cultured from the midgut of field-collected *Ae. aegypti* and *Ae. albopictus* and laboratory-reared *Ae. aegypti*.* FC-AEA* Field-collected *Ae. aegypti* adults,* FC-AEL* field-collected *Ae. aegypti* larvae,* FC-AAA* field-collected *Ae. albopictus* adults,* FC-AEL* field-collected *Ae. albopictus* larvae,* LC-AEA* laboratory-reared *Ae. aegypti* adults,* LC-AEL* laboratory-reared *Ae. aegypti* larvae

The phylogenetic distances estimated from *16S rRNA* gene sequences from bacteria isolated in this study placed *Elizabethkingia miricola* (the only species from phylum Bacteroidetes) on a long branch that seemed to cluster separately (Fig. 5). The phylum Actinobacteria, on the other hand, was very compact and contained very short branches. The phylum Firmicutes showed the most variation, with several distinct clusters. Genetic distance of two bacterial species (i.e. *Pantoea dispersa*, *Microbacterium paraoxydans*) differed between host mosquito species (*Ae. aegypti* and *Ae. albopictus*) from which they were isolated (Fig. 5).

## Discussion

The prevalence of mosquito gut microbial communities has been investigated previously using classical culture-based methods or by metagenomics using* 16S rRNA* gene sequencing [30–33]. Various factors may influence the midgut microbiota of mosquitoes, including geographical location [15]. For example, a study conducted in North America [34] on interactions between La Crosse virus and bacteria isolated from the digestive tract of *Ae. albopictus* revealed different species belonging to the genera *Erwinia*, *Vagococcus*, *Kluyvera*, *Pseudomonas*, *Chryseobacterium*, *Roseomonas*, *Pedobacter*, *Curtobacterium*, *Leuconostoc*, *Paenibacillus*, *Brenneria* and *Brenneria* [34], while another study found that *Ae. aegypti* from Panama harbored bacteria of the genera *Acetobacter*, *Bacillus*, *Staphylococcus*, *Enterobacter*, *Serratia* and *Pantoea* [35], which were also found in the present study.

The study reported here is the first of its kind in Sri Lanka. We found 25 species of midgut bacteria belonging to 14 genera. *Lysinibacillus sphaericus* was a common species in adults and larvae of laboratory-reared *Ae. aegypti.* Only *Pantoea dispersa* occurred in the field-collected adults of *Ae. aegypti* and *Ae. albopictus*. It has been suggested that the midgut bacterial diversity is acquired from various types of environments and also that diversity varies according to the life stage of mosquitoes [15]. In general, shifting of the feeding habits from high carbohydrate levels to proteins may elevate the level of enteric bacteria and thereby reduce overall bacterial diversity [36, 37].

The bacterial community in mosquito midguts of laboratory-reared mosquitoes is impacted by different feeding regimens used in different laboratories. A previous study revealed that host blood-meal source has a strong impact on gut microbiota of *Ae. aegypti* [38]. After sugar-feeding or blood-feeding, the gut bacterial diversity is known to decrease dramatically. Furthermore, two diet regimes tend to favor the proliferation of some bacterial taxa over others [38]. The metabolism of carbohydrate-rich sugars and protein-rich blood may create different gut conditions that may trigger the differential proliferation of bacterial taxa [13]. On the other hand, different host blood-meal types may also lead to the differential proliferation of microbial taxa in the mosquito gut, as different blood types vary with their total protein, hemoglobin and hematocrit content. However, the level of gut bacterial diversity can be restored to the original pre-blood meal levels once the blood meal is digested. Hence, in the selection of adult mosquitoes for midgut bacterial screening, we used non-blood-fed individuals.

In the present study, dissected midgut lysates were cultured to isolate the bacterial colonies, before *16S rRNA* gene sequencing. The main reason for using a traditional-based screening method rather than direct next-generation sequencing (NGS) was to isolate the bacterial species that can be cultured, which may be beneficial for paratransgenesis application if sufficient species with the required characteristics can be isolated [39, 40].

According to previous investigations, Actinobacteria and Bacteroidetes, members of Proteobacteria, were found to be consistently present in the larvae of both *Ae. aegypti* and *Ae. albopictus* [41–45]. Moreover, *Chryseobacteriuthm*, *Elizabethkingia*, *Pseudomonas*, *Nisseria*, *Microbacterium* and *Enterobacter* have also been frequently found in the gut of larvae of these mosquito species [11, 13, 33, 46–51]. In the present study, members of Proteobacteria, Actinobacteria and many species of Firmicutes were identified in *Ae. aegypti* and *Ae. albopictus*. Bacteria identified to species level belonged to the genera *Elizabethkingia*, *Nisseria*, and *Microbacterium*. Although *Chryseobacterium* (Flavobacteriaceae) has previously been found as a common component of mosquito microbiota at all life stages [33, 46–51], no species from this genus was recorded in the present study.

In adult mosquitoes, members of phyla Proteobacteria, Bacteroides, Firmicutes and Actinobacteria accounted for approximately 99% of the total microbiota community in previous studies [51], which is in line with our findings. More precisely, members of *Enterobacteriaceae* (e.g. *Enterobacter*), *Erwiniaceae* (e.g. *Pantoea*) and *Bacillaceae* (e.g. *Bacillus*) are the most frequently described bacteria from the gut adult *Aedes* spp. [12, 46, 52–59]. Our study also confirmed that bacteria from these genera predominated in both *Ae. aegypti* and *Ae. albopictus*. However, we detected bacteria of the genera *Terribacillus*, *Lysinibacillus*, *Agromyces* and *Kocuria* in larvae of both *Ae. aegypti* and *Ae. albopictus*, which have not been encountered from previous investigations.

Many studies have summarized the positive and negative effects of gut microbial communities on vector competency through host–parasite interactions [55, 58]. In addition, the midgut bacterial communities may secrete anti-viral metabolites. Three bacterial species isolated from *Ae. albopictus *in our study, namely *Enterobacter ludwigii*, *Pseudomonas rhodesiae* and *Vagococcus salmoninarium*, have been shown to inhibit La Crosse virus* in vitro* [34]. Therefore, their potential role in inhibiting the development of other viruses, such as DENV and chikungunya, in mosquito vectors should be assessed.

Under the paratransgenesis approach, *Bacillus megaterium* and *B. licheniformis* have been identified previously as suitable candidates for phlebotomine sand flies [60, 61]. In the present study, several *Bacillus* spp. were recorded, including *B. megaterium.* In addition, the present investigation identified *Lysinibacillus sphaericus*, which in a previous study had been used to modulate immunity/immune priming in mosquitoes and thereby to inhibit the development of malaria parasites in insect vectors [35]. Also, *Serratia odorifera* has been shown to enhance the viral infection in *Ae. aegypti* mosquitoes [62]. Although *S. odorifera* was was not observed in the current study, *S. liquefaciens* was a predominant species in laboratory-reared *Ae. aegypti* adults. According to a previous investigation, the genus *Pantoea* is a possible candidate for paratransgenesis, and it is known to influence the vector competence of *Ae. albopictus* as well [63]. *Pantoea* spp. exhibit transstadial and horizontal transmission properties [56]. *Pantoea agglomerans* has been shown to be able to express and secrete anti-*Plasmodium* effector proteins (SM1, anti-Pbs21, and PLA2), which can suppress malaria parasites in the mosquito vectors [64]. Hence, the possibility of using the recorded species of the genus *Pantoea* should be further evaluated. On the other hand, *Bacillus flexus*, *B. megaterium*, *B. nealsonii* and *Leucobacter chironomi* were recorded among laboratory-reared and field-collected *Ae. aegypti* and *Ae. albopictus*. Hence, it is essential to screen their suitability for use in a paratrangenesis-based vector control approach in Sri Lanka.

The results from this study augment current understanding of mosquito midgut bacteria and aid in curating microbiome data from susceptible and refractory *Aedes* spp. strains to identify factors shifting the balance toward mosquitoes that do not transmit arboviruses to humans. Overall, the present investigation illustrates the presence of midgut bacterial community in *Aedes* mosquitoes in selected areas that have been identified as operational sites for novel vector control strategies, such as SIT and IIT, in Sri Lanka.

There are a number of limitations to the study. The gut flora among insects is highly dynamic [65], which may have influenced the present findings. Only 20% of the bacteria in the gut environment can be grown on culture media according to the literature [61]. This is a limitation of the culture-dependent analysis, which does not allow an estimation of the whole gut bacterial community. The species-level characterization based on phylogenetic relationships of *16S rRNA* gene sequences may not be sufficiently precise for some bacterial genera. This is another limiting factor, even though this approach has been widely used for bacterial characterization [61, 66]. When possible, nucleic acid-based analysis, such as Sanger sequencing, automated ribosomal internal transcribed spacer analysis (ARISA), terminal restriction fragment length polymorphism (T-RFLP), denaturing gradient gel electrophoresis (DGGE), and NGS technology, should be used [61].

Overall, the present investigation provides the first attempt to document the presence of bacteria in the midgut of DENV vector mosquitoes in Sri Lanka. Despite the above-mentioned limitations, our results may motivate and encourage researchers to explore these aspects in Sri Lanka and widen the research capacity.

## Conclusions

This study generated a comprehensive database on the culturable bacterial community found in the midgut of field-collected (*Ae. aegypti* and *Ae. albopictus*) and laboratory-reared (*Ae. aegypti*) mosquito larvae and adults from Sri Lanka. Data confirm that the midgut bacterial diversity in the studied mosquitoes varies according to species, developmental stage and strain (field versus laboratory).

## Supplementary Information

**Additional file 1:Figure S1.** Primary culture plates of microbial colonies from field-collected *Aedes aegypti* adults. Colonies were grown sterile plate count agar at different dilutions.** a** 10:1,** b** 10:2,** c** 10:3.

**Additional file 2:Table S1.** List of gut bacterial species identified from field-collected and laboratory-reared adults and larvae of* Aedes* mosquitoes.

## Data Availability

The datasets supporting the conclusions of this article are included within the article and its additional files. All sequences generated in this study were deposited in GenBank and accession numbers are available in Additional file 2: Table S1.

## Abbreviations

ARISA: Automated ribosomal internal transcribed spacer analysis
BC: Bray–Curtis
BLAST: Basic Local Alignment Search Tool
CFUs: Colony-forming units
dbRDA: Distance-based redundancy analysis
DENV: Dengue virus
DGGE: Denaturing gradient gel electrophoresis
IIT: Insect incompatible technique
IVM: Integrated vector management
MOH: Medical Officer of Health
NGS: Next-generation sequencing
PBS: Phosphate-buffered saline
PCA: Plate count agar
SIT: Sterile insect technique
T-RFLP: Terminal restriction fragment length polymorphism
