# Supplementary figures and images for: Diversity of midgut bacteria in larvae and females of Aedes aegypti and Aedes albopictus from Gampaha District, Sri Lanka
Source: Parasit Vectors. 2021 Aug 28;14:433. doi: 10.1186/s13071-021-04900-5 (PMC8400895; doi:10.1186/s13071-021-04900-5)

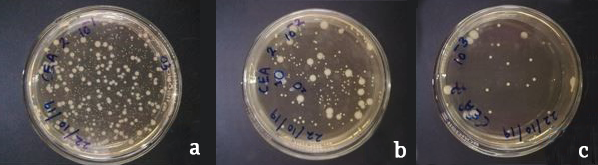

Supplement: Supplementary file 1 — Additional file 1:Figure S1. Primary culture plates of microbial colonies from field-collected Aedes aegypti adults. Colonies were grown sterile plate count agar at different dilutions. a 10:1, b 10:2, c 10:3. [file 13071_2021_4900_MOESM1_ESM.tif]
