# Supplementary material for: Diversity of midgut bacteria in larvae and females of Aedes aegypti and Aedes albopictus from Gampaha District, Sri Lanka
Source: Parasit Vectors. 2021 Aug 28;14:433. doi: 10.1186/s13071-021-04900-5 (PMC8400895; doi:10.1186/s13071-021-04900-5)
Supplement: Supplementary file 2 — Additional file 2:Table S1. List of gut bacterial species identified from field-collected and laboratory-reared adults and larvae of Aedes mosquitoes. [file 13071_2021_4900_MOESM2_ESM.docx]

**Additional file 2: Table S1.** List of gut bacterial species identified from field-collected and laboratory-reared adults and larvae of *Aedes* mosquitoes

| Phylum, family | Species | GenBank accession numbers | Percentage occurrence in mosquitoes midgut (95% CI) | | | | | |
| --- | --- | --- | --- | --- | --- | --- | --- | --- |
|  |  |  | FC-AEA | FC-AEL | FC-AAA | FC-AAL | LR-AEA | LR-AEL |
| Actinobacteria, *Microbacteriaceae* | *Agromyces* sp. | MT279343 | - | - | - | 10.1 (5.6-17.6) | - | - |
|  | *Leucobacter chironomi* | MT279355 | - | - | - | - | - | 28.1 (20.3-37.6) |
|  | *Microbacterium paraoxydans* | MT279356, MT279337 | - | - | - | 8.1 (4.2-15.1) | - | 17.2 (11.1-25.8) |
|  | *Microbacterium* sp. | MT279442, MT279341, MT279344 | - | - | - | 5.1 (2.2-11.2) | - | 7.8 (4.0-14.8) |
|  | *Microbacterium trichothecenolyticum* | MT275627 | - | - | 5.9 (2.7-12.3) | - | - | - |
| Actinobacteria, *Micrococcaceae* | *Kocuria kristinae* | MT275600 | - | - | 10.3 (5.7-17.8) | - | - | - |
| Bacteroidetes, *Flavobacteriaceae* | *Elizabethkingia miricola* |  | - | - | 22.1 (15.1-31.1) | - | - | - |
| Firmicutes, *Bacillaceae* | *Bacillus cereus* | MT279443, MT279464 | - | 4.7 (1.9-10.7) | - | - | - | 18.75 (12.3-27.5) |
|  | *Bacillus endophyticus* | MT277424 | 30.8 (22.6 40.4) | - | - | - | - | - |
|  | *Bacillus flexus* | MT279459, MT279468 | - | 27.9 (20.1-37.4) | - | - | - | - |
|  | *Bacillus megaterium* | MT279338 | - | - | - | 30.3 (22.2-39.9) | - | - |
|  | *Bacillus nanhaiensis* | MT279324, MT279329 | - | - | - | 25.3 (17.8-34.6) | - | - |
|  | *Bacillus nealsonii* | MT279461 | - | 18.6 (12.2-27.3) | - | - | - | - |
|  | *Bacillus* sp. | MT279460, MT279463, MT279327, MT279328, MT279345, MT279342 | - | 23.3 (16.1-32.4) | - | 21.21 (14.34-30.21) | - | - |
|  | *Lysinibacillus sphaericus* | MT279351, MT279353, MT279354, MT279445, MT279462 | - | 12.8 (7.6-20.7) | - | - | 35.5 (26.8-45.2) | 31.3 (23.0-40.9) |
|  | *Terribacillus* sp. | MT279473 | - | 8.1 (4.2-15.2) | - | - | - | - |
| Firmicutes, *Staphylococcaceae* | *Staphylococcus warneri* | MT277413 | 12.8 (7.6-20.8) | - | - | - | - | - |
|  | *Staphylococcus sciuri* | MT275460 | - | - | 14.7(9.9-19.1) | - | - | - |
| Proteobacteria, *Enterobacteriaceae* | *Enterobacter* sp. | MT277091, MT277412 | 20.5 (9.1-23.0) | - | - | - | - | - |
| Proteobacteria, Eriwiniaceae | *Pantoea dispersa* | MT275483, MT275806, MT275631, MT540024 | 20.5 (13.8-29.4) | - | 35.3 (26.6-45.0) | - | - | - |
| *Proteobacteria*, Moraxellaceae | *Acinetobacter baumannii* | MT277459 | 10.3 (5.7-17.8) | - | - | - | - | - |
|  | *Acinetobacter nosocomialis* | MT540255 | 5.1 (2.2-11.4) | - | - | - | - | - |
|  | *Acinetobacter* sp. | MT279465/ MT279467 | - | 4.7 (1.9-10.7) | - | - | - | - |
| *Proteobacteria*, Neisseriaceae | *Neisseria flavescens* |  | - | - | 11.8 (6.8-19.5) | - | - | - |
| Proteobacteria, Yersiniaceae | *Serratia liquefaciens* | MT279350 | - | - | - | - | 64.5 (54.8-73.2) | - |

^a^ All bacterial species were assigned to a species when the percentage identity was ≥ 99%.

*Abbreviations*: CI, confidence interval; FC-AEA, field-collected *Ae. aegypti* adults; FC-AEL, field-collected *Ae. aegypti* larvae; FC-AAA: field-collected *Ae. albopictus* adults; FC-AEL: field-collected *Ae. albopictus* larvae; LR-AEA, laboratory-reared *Ae. aegypti* adults; LR-AEL, laboratory-reared *Ae. aegypti* larvae.
